# Annual acknowledgement of manuscript reviewers

**DOI:** 10.1186/1471-230X-14-13

**Published:** 2014-01-17

**Authors:** Magdalena Morawska

**Affiliations:** 1 BioMed Central, Floor 6, 236 Gray's Inn Road, London WC1X 8HB, UK

## Abstract

**Contributing reviewers:**

The editors of *BMC Gastroenterology* would like to thank all of our reviewers who have contributed to the journal in Volume 13 (2013).

## 

Aaron Thrift

Australia

Abbi Saniabadi

Japan

Abdulrasheed Nasir

Nigeria

Adam Weizman

Canada

Adarsh Chaudhary

India

Adedayo Onitilo

USA

Agostino Di Ciaula

Italy

Ahmet Ozer Sehirli

Turkey

Aijaz Sofi

USA

Ajith Siriwardena

UK

Akif Altinbas

Turkey

Akira Horiuchi

Japan

Alain Demers

Canada

Alain Gobert

France

Alan Moss

USA

Alberto Vannelli

Italy

Albrecht Hoffmeister

Germany

Alcides Chaux

Paraguay

Aleksandar Nagorni

Serbia

Alessio Aghemo

Italy

Alessio Pini Prato

Italy

Alexander Wree

USA

Alexander Koch

Germany

Alexander Link

Germany

Alexandra Meziti

Greece

Alfred Cheng

Hong Kong

Alfreda Krupoves

Canada

Alfredo J Lucendo

Spain

Ali Canbay

Germany

Alisan Kahraman

Germany

Aliye Esmaoglu

Turkey

Allauddin Siddiqi

New Zealand

Amit Maydeo

India

Amitabh Suman

USA

Ana Lleo

Italy

Anastasia Konidari

UK

Anastasios Koulaouzidis

UK

Anders Rönnblom

Sweden

Andrea Zanichelli

Italy

Andreas Mentis

Greece

Andreas Weber

Germany

Andrew Day

Australia

Andrew Slater

UK

Andrew Hart

UK

Andrew Veitch

UK

Angelo Zullo

Italy

Angelo Di Giorgio

Italy

Anja Geerts

Belgium

Anna Myleus

Sweden

Anna Mokrowiecka

Poland

Anna Pecorelli

Italy

Anna Sapone

Italy

Anne Salonen

Finland

Annie Anderson

UK

Ans Pauwels

Belgium

Anshu Srivastava

India

Antongiulio Faggiano

Italy

Antoni Stadnicki

Poland

Antonio Ascione

Italy

Antonio Tursi

Italy

Antonio Fontanellas

Spain

Antonio Di Sabatino

Italy

Antonio Gasbarrini

Italy

Antonio Martín Maya

Argentina

Antti Koivusalo

Finland

Anubhav Mittal

Australia

Ardem Patapoutian

USA

Arjun Koch

Netherlands

Arjuna De Silva

Sri Lanka

Armin Alaedini

USA

Arnaldo Amato

Italy

Arne Kandulski

Germany

Arun Swaminath

USA

Arunkumar Krishnan

India

Ashish Chintakuntlawar

USA

Ashok Saluja

USA

Ashraf Mohabati Mobarez

Iran

Ashwin Ananthakrishnan

USA

Atsushi Yoden

Japan

Atsushi Nakajima

Japan

Aurelie Plessier

France

Avdyl Krasniqi

Serbia

Balakrishnan S Ramakrishna

India

Beat Schnüriger

Switzerland

Bernhard Watzl

Germany

Biguang Tuo

China

Biljana Mihaljevic

Serbia

Bjorn Moum

Norway

Bo Shen

USA

Bolette Hartmann

Denmark

Bonnard Arnaud

France

Brad Confer

USA

Brendan Jenkins

Australia

Brian Bressler

Canada

Brian Perrino

USA

Brian Yan

Canada

Bridget Southwell

Australia

Bruna Roesler

Brazil

Bruno Rosa

Portugal

Bruno Bonaz

France

Byung Lee

South Korea

Byung Ihn Choi

South Korea

Caiyan Zhao

China

Camilla Nøjgaard

Denmark

Carl Johan Wingren

Sweden

Carlo Selmi

USA

Carlo Schneider

Germany

Carlo Catassi

Italy

Carlos Areche

Chile

Carol Gardner

USA

Carol Burke

USA

Carole Brasse-Llagnel

France

Carsten Schmidt

Germany

Cesare Cremon

Italy

Chai Soon Ngiu

Malaysia

Chang-Ming Huang

China

Chao-Hung Hung

Taiwan

Charalabos Pothoulakis

USA

Charles Levea

USA

Charles Cox

USA

Charles Bernstein

Canada

Chien-Yun Hsiang

Taiwan

Chihiro Sasakawa

Japan

Chris Dekaney

USA

Chris Rayner

Australia

Christian Fingas

Germany

Christian Stock

Germany

Christian Jenssen

Germany

Christina Ha

USA

Christoph Röcken

Germany

Christoph Jochum

Germany

Christoph Neumann-Haefelin

Germany

Christophe Mariette

France

Christopher Bowlus

USA

Christopher Anderson

USA

Christopher Schalchta

Canada

Chrong-Reen Wang

Taiwan

Chuansheng Wang

USA

Chun-Jen Liu

Taiwan

Claudio Puoti

Italy

Claudio Sette

Italy

Claus Fimmel

USA

Cord Langner

Austria

Craig Friesen

USA

Cuneyt Kayaalp

Turkey

Daniel Leske

Germany

Daniel Reim

South Korea

Daniel Barry

USA

Daniel Antoine

UK

Daniela Cabibi

Italy

Daniela Jakubowicz

Israel

Danyvid Olivares-Villagomez

USA

Dara Kavanagh

Ireland

David Watson

Australia

David Scholten

Germany

David Morris

Australia

David Wang

USA

David Chang

UK

David Back

UK

David Sass

USA

David Goldstein

Australia

Davide Festi

Italy

Debbie Shawcross

UK

Deborah Stearns-Kurosawa

USA

Decker Butzner

Canada

Deepak Agrawal

USA

Deng-Chyang Wu

Taiwan

Dennis Ougrin

UK

Derek O'Reilly

UK

Devendra Amre

Canada

Devinder Dhawan

India

Dhira Saha

India

Diana Karpman

Sweden

Dina Tiniakos

Greece

Dino Vaira

Italy

Ditlev Nytoft Rasmussen

Denmark

Do Youn Park

South Korea

Don W Powell

USA

Donald O Castell

USA

Donat Rudolf Spahn

Switzerland

Eamonn Quigley

Ireland

Earl Williams

UK

Eckart Schott

Germany

Edgardo Smecuol

Argentina

Edoardo Savarino

Italy

Efimia Papadopoulou-Alataki

Greece

Efthymios Avgerinos

USA

Eithne Costello

UK

Elizabeth Erwin

USA

Elke Eggenhofer

Germany

Emanuel Manesis

Greece

Emmanuel Tsochatzis

Greece

Enrique Rey

Spain

Erik Solligard

Norway

Erkan Parlak

Turkey

Erwin Biecker

Germany

Eva Szigethy

USA

Eva Herrmann

Germany

Ewa Ignatowicz

Poland

Ezgi Gulmez

France

Fabian Schnitzler

Germany

Fabiana Zingone

Italy

Fabio Pace

Italy

Fabrizio Bronte

Italy

Fadi Rzouq

USA

Faouzi Saliba

France

Fausto Catena

Italy

Federico Biagi

Italy

Fei Bao

USA

Fekicity Eb May

UK

Felice O'Ryan

USA

Felix Leung

USA

Fenghong Huang

China

Ferenc Sipos

Hungary

Fermin Sanchez De Medina

Spain

Fernando Gomollón García

Spain

Fernando Fornari

Brazil

Filippo Catalano

Italy

Filippo Cremonini

USA

Francesco Valitutti

Italy

Francis Vasseur

France

Francois Blachier

France

Frank CM Kneepkens

Netherlands

Frank Tacke

Germany

Frank Friedenberg

USA

Frauke Stanke

Germany

Fredrik Norström

Sweden

G Kang

India

Gabrio Bassotti

Italy

Garth Utter

USA

Geoffrey Holmes

UK

George Therapondos

USA

Georgina Hold

UK

Giammaria Fiorentini

Italy

Gian Andrea Binda

Italy

Gian Luigi De' Angelis

Italy

Gianluca Perseghin

Italy

Giovanni Galati

Italy

Giovanni Carini

Italy

Giovanni Di Nardo

Italy

Giuseppe Fede

Italy

Giuseppe Cabibbo

Italy

Gi-Young Ko

South Korea

Gokulakrishnan Kuppan

India

Gourdas Choudhuri

India

Govind Makharia

India

Grant Sanders

UK

Greger Lindberg

Sweden

Guillermo Ignacio Perez-Perez

USA

Guoxin Li

China

Gyeong Hoon Kang

South Korea

Hans Orlent

Belgium

Hans Herfarth

USA

Harkirat Singh

USA

Harry Xia

USA

Hartmut Jaeschke

USA

Heike Bantel

Germany

Heikki Jarvinen

Finland

Heinz Wykypiel

Austria

Helmut Denk

Austria

Hendrik Manner

Germany

Henning Wittenburg

Germany

Henri Braat

Netherlands

Henri Leuvenink

Netherlands

Henrik Koehler

Germany

Heshaam Mir

USA

Hideaki Miura

Japan

Hien Huynh

Canada

Hirofumi Uto

Japan

Hiroki Endo

Japan

Hiroki Yamaue

Japan

Hironori Mitsuyoshi

Japan

Hiroomi Okuyama

Japan

Hiroyuki Kirikoshi

Japan

Holger Lutz

Germany

Holly Algood

USA

Hossein Ali Arab

Iran

Hua-Chuan Zheng

China

Hulya Aksoy

Turkey

Ian Campbell

Australia

Ihsan Ekin Demir

Germany

Ilyssa Gordon

USA

Imran Aziz

UK

Ingrid Lisanne Holster

Netherlands

Inti Zlobec

Switzerland

Isabella Inden

Germany

Itoh Yoshito

Japan

Ivan Hung

Hong Kong

Jakob Izbicki

Germany

James Franciosi

USA

James O'Beirne

UK

James L Smith

USA

Jan Bornschein

Germany

Jan Preiss

Germany

Jan Best

Germany

Jan Gunnar Hatlebakk

Norway

Jan-Werner Poley

Netherlands

Jeffrey TJ Huang

UK

Jennifer Kramer

USA

Jennifer Vittorio

USA

Jennifer Debruyn

Canada

Jeremy French

UK

Jeremy Woodward

UK

Jesse Lachter

Israel

Jing-Yu Deng

China

Jing-Yuan Fang

China

Joao Sousa

Brazil

Jochen Schuld

Germany

Joel Lavine

USA

Joel Mawdsley

UK

Joern M Schattenberg

Germany

Johannes Jeekel

Netherlands Antilles

Johannes Wiegand

Germany

Johannes Hadem

Germany

John Mastronarde

USA

John Dillon

UK

John Clancy

USA

John Triantafillidis

Greece

Jon Walker

USA

Jonas Ludvigsson

Sweden

Jonas Adrian Scheiber

Germany

Jonel Trebicka

Germany

Jorgen Agnholt

Denmark

Jorgen Olsen

Denmark

Jorma Halttunen

Finland

Jose Pajares

Spain

Jose Ignacio Herrero

Spain

Juan Caballeria

Spain

Juan R Larrubia

Spain

Jude Oben

UK

Juei-Tang Cheng

Taiwan

Julia Kälsch

Germany

Julio Galvez

Spain

Julius Spicak

Czech Republic

Junfang Ji

USA

Junko Aida

Japan

Juozas Kupcinskas

Lithuania

Jürgen Scheller

Germany

Kalle Kurppa

Finland

Kamran Rostami

UK

Karel Geboes

Belgium

Karen Makar

USA

Karen Bronner

Israel

Karsten Schulmann

Germany

Karsten Wursthorn

Germany

Kartar Singh

India

Katarzyna Kilis-Pstrusinska

Poland

Katherine Mcglynn

USA

Katsunori Iijima

Japan

Katsutaka Oishi

Japan

Kazuhiro Tsuruma

Japan

Kazumasa Miyake

Japan

Kazuo Hara

Japan

Keisuke Hori

Japan

Keliang Xie

China

Kenan Kaygusuz

Turkey

Kentaro Sugano

Japan

Kevin Reavis

USA

Kirsteen Browning

USA

Klaus Herrlinger

Germany

Kong Boo Phua

Singapore

Konrad Streetz

Germany

Krzysztof Celinski

Poland

Kshipra Singh

USA

Kt Park

USA

Kyoung Kim

South Korea

Lani Prideaux

Australia

Larissa Fujii

USA

Lars Stenhammar

Sweden

Lars Aabakken

Norway

László Herszényi

Hungary

Laurence Bouillet

France

Laurent Peyrin-Biroulet

France

Lei Lian

China

Lene Hjerrild Iversen

Denmark

Leonardo Henry Eusebi

Italy

Leticia Moreira

Spain

Li Liang

China

Li Ning

China

Liam Murray

UK

Lian-Jie Lin

China

Lise Gluud

Denmark

Lixia Xu

Hong Kong

Lori Fischbach

USA

Lori Coburn

USA

Lorraine Chantrill

Australia

Louis Valiquette

Canada

Lubka Roumenina

France

Lucinda Harris

USA

Lyudmila Boyanova

Bulgaria

Malaisamy Muniyandi

India

Malay Sharma

India

Mandana Rafeey

Iran

Mangesh Pagadala

USA

Manlio Vinciguerra

UK

Manon Spaander

Netherlands

Manuel Barreiro De Acosta

Spain

Manuele Furnari

Italy

Marcel Spanier

Netherlands

Marcella Arru

Italy

Marcello Fabio Maida

Italy

Marcin Polkowski

Poland

Marcis Leja

Latvia

Marco Montorsi

Italy

Marco Di Girolamo

Italy

Marek Brabec

Czech Republic

Maria Pellise

Spain

Maria Pozo

Spain

Maria Tsolia

Greece

Maria Kalafateli

Greece

Maria Roubelakis

Greece

Maria Piazuelo

USA

Mária Papp

Hungary

Maria Antonietta Mazzei

Italy

Maria Francisca Gonzalez-Escribano

Spain

Mariagrazia Rumi

Italy

Mariana Leal

Brazil

Marie-Hélène Vieillard

France

Marina Silveira

USA

Mario Mondelli

Italy

Marjolijn Duijvestein

Netherlands

Mark Wulkan

USA

Markku Nissinen

Finland

Marko Kalliomäki

Finland

Martijn Bauer

Netherlands

Martin Gramatzki

Germany

Martin Hug

Germany

Masaaki Kobayashi

Japan

Masaaki Kodama

Japan

Masakuni Kobayashi

Japan

Masami Omae

Japan

Masayuki Kitano

Japan

Massimo Bellini

Italy

Mate Knabe

Germany

Mathewos Tessema

USA

Mathieu Piche

Canada

Matteo Fassan

Italy

Matthew Kurien

UK

Matthew Carroll

Canada

Matthew Cave

USA

Matthias Zilbauer

UK

Maurizio Koch

Italy

Mehmet Yilmaz

Turkey

Mercedes Iñarrairaegui

Spain

Mi Jeong Sung

South Korea

Michael Pitiakoudis

Greece

Michael Moser

Canada

Michael Zasloff

USA

Michael Selgrad

Germany

Michael Rünzi

Germany

Michael Miller

UK

Michael Wargovich

USA

Michael Crawford

Australia

Michael Blennerhassett

Canada

Michael Linnebacher

Germany

Michael Vieth

Germany

Michele Malaguarnera

Italy

Michele Amoruso

Italy

Michitaka Suzuki

Japan

Miika Arvonen

Finland

Min Fang

China

Mireen Friedrich-Rust

Germany

Mirella Fraquelli

Jamaica

Mohamad Imam

USA

Mohamed Hebbar

France

Mohammad Khuroo

India

Mohammad Abdollahi

Iran

Mohammad Derakhshan

UK

Moïse Coëffier

France

Morichika Konishi

Japan

Morten Würtz

Denmark

Morten Helms

Denmark

Motohiro Esaki

Japan

Motoyuki Kohjima

Japan

Munechika Enjoji

Japan

Myung-Hwan Kim

South Korea

Nadeem Siddiqui

Pakistan

Nanne De Boer

Netherlands

Naoki Oishi

Japan

Narci Teoh

Australia

Natalia Osna

USA

Nathan Johnson

Australia

Nayoung Kim

South Korea

Nazri Mustaffa

Malaysia

Neil Shah

UK

Neil Gerard Docherty

Ireland

Niall Patrick Hyland

Ireland

Nicola De Bortoli

Italy

Nigel Bird

UK

Nina Kimer

Denmark

Nina Weis

Denmark

Nobuyuki Hamajima

Japan

Nobuyuki Matsuhashi

Japan

Nonthalee Pausawasdi

Thailand

Norbert Cibicek

Czech Republic

Norberto Chavez-Tapia

Mexico

Norifumi Harimoto

Japan

Nuruddeen Lewis

USA

Oliver Schwandner

Germany

Olivera Stanojlovic

Serbia

Oliviu Pascu

Romania

Olof Sandström

Sweden

Olympia Anastasiou

Germany

Omar Hyder

USA

Osamu Dohi

Japan

Osamu Inatomi

Japan

Össur Ingi Emilsson

Iceland

Pamela Lochhead

UK

Pamela Minicozzi

Italy

Pandu Gangula

USA

Paola Iovino

Italy

Parakkal Deepak

USA

Pasquale Innominato

France

Patricia Sancho

Spain

Patrick Michl

Germany

Patrizia Ferroni

Italy

Paul Drew

Australia

Pavel Strnad

Germany

Pavol Papay

Austria

Pedram Ghafourifar

USA

Per Pfeiffer

Denmark

Pere Clave

Spain

Peter Green

USA

Peter Malfertheiner

Germany

Peter Hauser

USA

Peter Reismann

Hungary

Peter Mills

UK

Petr Ricanek

Norway

Petr Husa

Czech Republic

Petra Ina Pfefferle

Germany

Petros Zezos

Greece

Philipp Lenz

Germany

Philippe Lehours

France

Philippe Mathurin

France

Philippe Marteau

France

Piero Chirletti

Italy

Piero Luigi Almasio

Italy

Pierre Nahon

France

Pilar Garcia-Peñarrubia

Spain

Pirjo Wacklin

Finland

Prakash C Gyawali

USA

Preet Paul Singh

USA

Prithviraj Rao

UK

Priyanka Trivedi

India

Puja Myles

UK

Qayyim Said

USA

Rachele Ciccocioppo

Italy

Raffaele Pezzilli

Italy

Raffaella Nenna

Italy

Raj Aravalli

USA

Rajinder Dawra

USA

Rakesh Kochhar

India

Ralf Weiskirchen

Germany

Raluca Pais

France

Rani Kanthan

Canada

Rebekka Schneider-Kramann

Germany

Reino Pöyhiä

Finland

Renumathy Dhanasekaran

USA

Reza Malekzadeh

Iran

Rhonda Yantiss

USA

Riad Rahhal

USA

Richard Douard

France

Richard Stevens

USA

Richard Ten Broek

Netherlands

Richelle Jeannette Fyline Felt

Netherlands

Risto Roine

Finland

Robert Roth

USA

Robert Martin

USA

Robert De Knegt

Netherlands

Robert Thimme

Germany

Rodrigo Machado

Brazil

Roja Rahimi

Iran

Romaric Loffroy

France

Rommel Burbano

Brazil

Rosana Risques

USA

Roy Sherwood

UK

Rui Manuel Reis

Portugal

Runhua Shi

USA

Rupesh Chaturvedi

USA

Rupjyoti Talukdar

India

Ryuichi Iwakiri

Japan

Sadegh Massarrat

Iran

Salah Aref

Egypt

Salah Mahmud

Canada

Saleem Islam

USA

Salvatore Gruttadauria

Italy

Salvatore Petta

Italy

Samad Amini-Bavil-Olyaee

USA

Samuel Andreas Käser

Switzerland

Sanjay Marwah

India

Sanjiv Mahadeva

Malaysia

Sarah Ballou

USA

Satoshi Ono

Japan

Satoshi Yamashita

Japan

Saurabh Chawla

USA

Savino Bruno

Italy

Sean Candrilli

USA

Serena Schippa

Italy

Sergei Grivennikov

USA

Sergio Cadoni

Italy

Seth Sweetser

USA

Shahid Umar

USA

Shahin Shafiani

USA

Shailender Singh

USA

Shi Yang

USA

Shree R. Singh

USA

Shuguang Wang

China

Shuji Ogino

USA

Shunji Fujimori

Japan

Shunsuke Nojiri

Japan

Shu-Yuan Xiao

China

Siddharth Singh

USA

Siew Ng

Hong Kong

Silvia Fargion

Italy

Silvia Melgar

Ireland

Siobhain O' Mahony

Ireland

Slobodan Tanaskovic

Serbia

Songyuan Tang

China

Sonia Michail

USA

Srinivasan Dasarathy

USA

Stefan Rauch

Germany

Stefan Acosta

Sweden

Stefania Mondello

USA

Stefania De Lisi

Italy

Stefano Trastulli

Italy

Stefano Fiorucci

Italy

Stefano Guandalini

USA

Steffen Zopf

Germany

Stella Smith

Nigeria

Stephan C Bischoff

Germany

Stephen Pereira

UK

Steven Werlin

USA

Stuart Mcdonald

UK

Sudeep Khanna

India

Sumiya Ishigami

Japan

Sung Joon Kwon

South Korea

Sunny Hei Wong

Hong Kong

Susana Fuentes

Netherlands

Susanne Hempel

USA

Susumu Eguchi

Japan

Svenja Sydor

Germany

Tae Hoon Lee

South Korea

Taichiro Nishikawa

Japan

Takafumi Ando

Japan

Takafumi Tabuchi

Japan

Takashi Kishimoto

Japan

Takayuki Yoshino

Japan

Tao Luo

China

Tarun Rai

USA

Teerha Piratvisuth

Thailand

Teh-Ia Huo

Taiwan

Teri Longacre

USA

Terumi Kamisawa

Japan

Tetsuji Fujita

Japan

Tetsuo Takehara

Japan

Theodore Liakakos

Greece

Thomas Chang

Canada

Thomas Wex

Germany

Thomas Schreiter

Germany

Tiberiu Hershcovici

USA

Timothy Price

Australia

Tom Bates

UK

Tom Moreels

Belgium

Tony Bruns

Germany

Toru Akiyama

Japan

Toru Ishikawa

Japan

Toshio Watanabe

Japan

Toshnari Takamura

Japan

Tsung-Teh Wu

USA

Uday Ghoshal

India

Ulf Herbers

Germany

Ulf Elbelt

Germany

Ulrich Keilholz

Germany

Umberto Volta

Italy

Umberto Vespasiani-Gentilucci

Italy

Upender Manne

USA

Uta Dahmen

Germany

Veronica Salvatore

Italy

Victor Moreno

Spain

Victor Cardenas

USA

Vincent Zimmer

Germany

Vincenza Calvaruso

Italy

Vincenzo De Francesco

Italy

Virendra Singh

India

Wei Gong

China

Weimin Ye

Sweden

Wen-Huang Peng

Taiwan

Wenjuan He

USA

Werner Draaisma

Netherlands

Wieslaw Bochenek

USA

Wijnand Laan

Netherlands

Wim Derave

Belgium

Wim Laleman

Belgium

Wolfgang Jugner

USA

Wolfgang Kratzer

Germany

Xavier Roblin

France

Xianrui Wu

China

Xuelong Zhang

China

Yaping Tian

China

Yasuhiro Asahina

Japan

Yasuo Suzuki

Japan

Yehuda Shoenfeld

Israel

Yeong Yeh Lee

Malaysia

Yi Ba

China

Yinghong Wang

USA

Yingqun Wang

USA

Yolanda Pacheci

Spain

Yong Sung Kim

South Korea

Yoon Jun Kim

South Korea

Yoshiaki Arimura

Japan

Yoshikatsu Koga

Japan

Yoshiko Nakayama

Japan

Yoshio Sumida

Japan

Yoshiro Aoki

Japan

Young-Tae Bak

South Korea

Yueyong Liu

USA

Yuji Naito

Japan

Yuri Saito

USA

Yusuke Okuyama

Japan

Yusuke Nishizawa

Japan

Yvette Taché

USA

Yvette Leung

Canada

Zheng Chen

China

Zheng-Feng Yin

China

Zsófia Küronya

Hungary

## Pre-publication history

The pre-publication history for this paper can be accessed here:

http://www.biomedcentral.com/1471-230X/14/13/prepub

